# Prevalence of psychiatric diagnoses in asylum seekers with follow-up

**DOI:** 10.1186/s12888-018-1783-y

**Published:** 2018-06-20

**Authors:** Kneginja Richter, Lukas Peter, Hartmut Lehfeld, Harald Zäske, Salina Brar-Reissinger, Günter Niklewski

**Affiliations:** 1 Clinic Nuremberg, University Clinic for Psychiatry and Psychotherapy, Paracelsus Private Medical University, Nuremberg, Germany; 2 Faculty for Social Sciences, Technical University for Applied Sciences Georg Simon Ohm, Nuremberg, Germany; 3 Faculty for Medical Sciences, University Goce Delcev, Stip, Macedonia; 4 Department of Psychiatry and Psychotherapy, Medical Faculty, Heinrich-Heine-University, Düsseldorf, Germany

**Keywords:** Asylum seekers, Refugees, Mental health, PTSD, Depression, Insomnia

## Abstract

**Background:**

In the study, the frequency and nature of asylum seekers’ psychiatric diagnoses in a German admission center were examined. Additional aims were to identify changes in those diagnoses over time and to investigate health care utilization of mentally ill asylum seekers in the community.

**Methods:**

The sample for the study “Psychiatric Examination of Asylum Seekers” in Bavaria consisted of a total of 283 asylum seekers and included 2 subsamples: help-seeking individuals and a randomly selected group. 34 of all asylum seekers were part of an extensive psychiatric follow-up examination (t2) about six months after the first examination (t1). Here, we used psychometric tools and a psychiatric interview by a medical doctor and a psychologist with the help of translators.

**Results:**

79% of help-seekers and 45% of the random group received at least one psychiatric diagnosis at t1. The most frequent diagnoses were trauma- and stress-related disorders, affective disorders, and insomnia. Men and Muslims were underrepresented in the help-seeking group. In the follow-up subsample, the rate of psychiatric diagnoses went down from 74% at t1 to 38% at t2. In contrast, the number of PTSD cases increased from 4 at t1 to 7 at t2. The severity of PTSD symptoms such as hyperarousal and avoidance also increased. Of the 13 persons in the follow-up-sample diagnosed with depression at t1, only 2 still fit the criteria of the disease at t2. Only 5 subjects had received some sort of psychotherapy or counseling.

**Conclusion:**

The prevalence of mental illness in asylum seekers reported here corresponds to the usual range in the literature. It is significantly higher than in European civil society, especially regarding PTSD. At t2, the diagnoses of PTDS had increased within several months without evident additional traumatic events. Asylum seekers’ psychiatric diagnoses soon after arrival should be recorded carefully and examination should be repeated after six months. The psychiatric and psychotherapeutic treatment of asylum seekers is still insufficient. Psychoeducative steps should be taken to relieve the stigma on mental illness, especially among males and Muslims.

## Background

Asylum seekers are among the most vulnerable, marginalized and powerless groups in society with a high risk for psychiatric disturbances. They have most likely only recently fled difficult living conditions [1–8] and now live in constant fear of deportation and under permanent strain in admission centers, which contributes to their psychological distress.

Germany was the country with the highest number of applications for asylum (65,000) within the European Union in the Year 2012, recording an increase of 41% in application numbers as compared to 2011 [9].

This study was conducted in one of Bavaria’s biggest admission centers, the Admission Center for Asylum Seekers (ZAE) – Zirndorf. The goals were to examine asylum seekers’ psychiatric diagnoses in the first weeks after their arrival in the country, to verify the diagnoses a few months after their discharge from the center and to examine whether mentally ill asylum seekers had received appropriate psychiatric and psychological treatment after discharge.

Most studies on mental disorders in asylum seekers focus on posttraumatic stress disorder (PTSD), reporting a range of prevalence rates between 32 and 46% [4, 10–16]. Others report numbers as high as 74% [17] or as low as 14% [18]. Affective and anxiety disorders were found to be the most frequent comorbidities with PTSD [19–21].

A review of published studies on the prevalence of mental disorders in asylum seekers shows a fundamental problem which makes a comparison of results difficult: the sampling methods used are very heterogeneous and led to samples that range from a randomly selected group of subjects to an examination of a rigidly selected group such as the population using counselling or other services [22].

Trying to build on and extend the reviewed research, we formulated the following questions: What are the prevalence rates of mental illness in groups of randomly selected and help-seeking asylum seekers? Which sociodemographic groups will be represented in these two groups? How will psychiatric diagnoses develop over time? And finally, in what ways will mentally ill asylum seekers receive treatment in the German public health system?

## Methods

Within the project “Psychiatric Examination of Asylum Seekers” supported by the Bavarian Ministry of Labour, Social Affairs, Family and Women [23], a psychiatric center was installed in an admission center in southern Germany. Here, two samples of asylum seekers underwent examination for mental disorders in a preliminary study (t1) between 01/2011 and 05/2012. The first sample consisted of help-seekers, i.e. individuals who had contacted the center of their own impetus. Information regarding psychiatric services was freely available to the residents of the admission center in the form of flyers which had been translated into ten different languages. As a result of close working relationships with local services, medical practitioners, and volunteer services, the project coordinators were able to widely disseminate information about the psychiatric services offered. The random sample consisted of randomly selected residents of the center. As part of the randomization process, a cross-check procedure was used to ensure that no participants from the help seeking group were invited to the random group. All of the invited individuals agreed to participate in the initial screening and all participants provided their consent in written form. Following written agreement from the Ethical Committee, participants were interviewed within the first six weeks of their residence in the center. The most common countries of origin in both samples were Iran (30.4%), Afghanistan (20.1%), Russia (19.4%), Iraq (14.5%), and Azerbaijan (5.7%). Islam (66.5%) and Christianity (13.9%) were the most common religious denominations.

Participants were interviewed by trained bilingual specialists who administered questionnaires to assess the frequency of mental disorders in the 12 months before the interview. Where possible, patients’ preferences with regard to the examiner’s gender were accommodated. Diagnoses were made in accordance with the International Classification of Diseases, 10th revision (ICD 10; [24]) using the Mini-International Neuropsychiatric Interview (MINI; [25]). We assessed psychiatric symptoms by use of the Brief Symptom Inventory (BSI), a culturally sensitive 53-item self-report symptom checklist. Based on BSI results, nine primary symptom dimensions can be computed: somatic symptoms, obsessive-compulsive symptoms, feelings of personal inadequacy, depression, clinical anxiety, anger and hostility, phobic anxiety, paranoid ideation, and social alienation [26]. To measure suicidal thoughts, item 10 of the Montgomery Asberg Depression Rating Scale (MADRS) was used, explicitly asking on a seven point scale for the feeling that life is not worth living, death would be welcome, suicidal thoughts are present or that a suicidal act is being prepared [27]. PTSD symptoms were measured using the Essen Trauma Inventory (ETI), a screening inventory consisting of 35 items for a broad range of traumatic events and 23 symptom based items for posttraumatic stress symptoms on four subscales: intrusion, avoidance, hyperarousal, and peritraumatic dissociation [28]. To assess sleep disturbances, we used the Pittsburg Sleep Quality Index (PSQI), concentrating on the first three of its seven subscores, i.e. subjective sleep quality, sleep latency, and sleep duration [29]. In addition, the interview included questions about the asylum seekers’ use of health services, as well as on social, economic and cultural factors since the migration.

The examinations were performed over a 3-h period during which a psychiatric history was taken by a psychiatry specialist and psychometric testing was carried out by a psychologist. The psychologist/psychiatrist provided detailed information to the participants on the background of the study and the examination procedure. In all cases, the psychiatric-psychological examination was carried out fully after the person selected for examination had given their written consent.

**Fig. 1:**
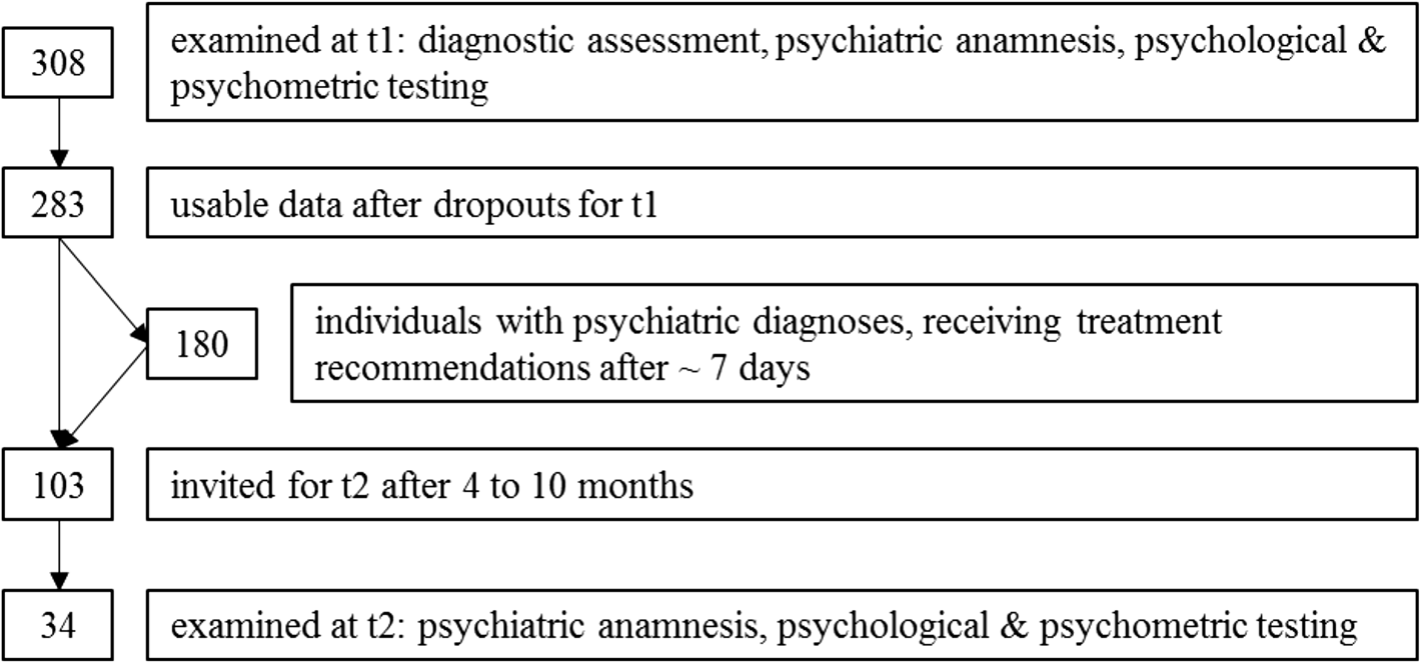
Steps of examination

One week after the first interview, subjects that had received a psychiatric diagnosis were asked to return for a second interview (see fig. 1). In this second consultation, therapeutic recommendations for future treatment were made after a discussion of the results and the diagnosis with the patient. Persons without a psychiatric diagnosis were not invited to the second interview.

Four to ten months later, after the asylum seekers had been discharged from the admission center to steadier accommodations, a follow-up examination (t2) was carried out. Our aim was to interview subsamples of both initially examined groups in their new homes. At t2, most individuals in the sample were either of Iranian (44.1%), Afghan (26.5%), or Russian (11.8%) nationality.

Statistical Analyses were performed using the program SPSS 20. Chi-square tests and variance analysis methods were used for interference statistical comparisons of the two subsamples in addition to the descriptive presentation of results by frequency charts and mean values. Missing data was excluded from the statistical analysis.

## Results

### Preliminary examination (t1)

**Fig. 2:**
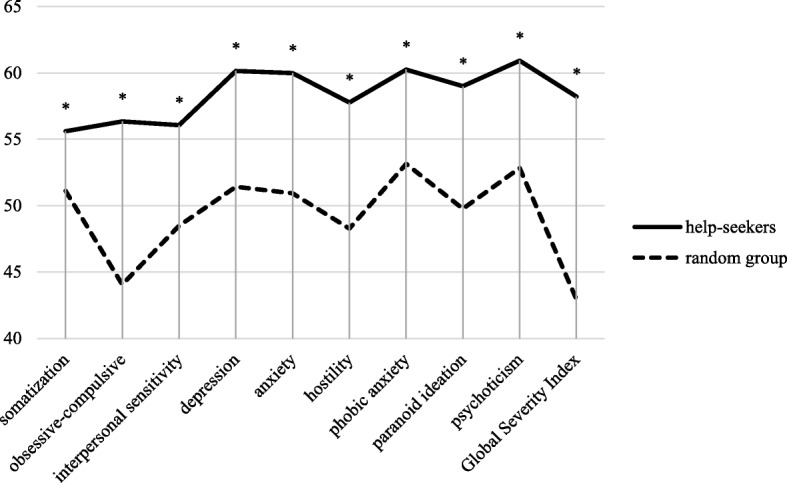
BSI profile plots for both groups at t1 (t-scores). *N* = 283. All differences *p* < .000

In total, 308 individuals were examined. After dropouts, data for 283 asylum seekers (125 women, 158 men, age 31.9 ± 10.6 years) was analysed (see fig. 2). The most common reasons for dropout were fatigue or decompensation during the examination and refusal of the psychological examination after the psychiatric interview. 158 of the examined individuals were part of the help-seeking sample, 125 formed the random group.

Women (51.9% vs. 34.4%, Chi-square test *p* < .01), Christians (22.4% vs. 17.7%, *p* < .05), Russians (27.8% vs 8.8%, *p* < .000) and Afghans (24.1% vs. 15.2%, *p* < .000), as well as those with previous migration experience (13.9% vs. 1.6%, *p* < .001) were significantly overrepresented in the help-seeking group as compared to the random group. We also found a tendency of those arriving with children prevailing among help-seekers (55.1% vs. 44.8%, *p* = .088), most likely because of the higher amount of women in this group. Men (48.1% vs 65.5%; *p* < .01) and Muslims (50.4% vs. 66.5%; *p* < .05) were underrepresented among help-seekers. There were no significant differences regarding age, marital status, education, or professional status.

Significantly more psychiatric diagnoses were made in the help-seeking group than in the random group (78.5% vs. 44.8%, *p* < .000, see Table 1). The most common primary diagnoses were stress-related and anxiety disorders (F4 and F3; 53.3% of diagnoses), affective disorders (34.4%) and insomnia (7.2%). There was no significant difference between the groups in the relative frequency of diagnoses (see Table 2). The most frequent diagnosis was PTSD with 17.6% of all subjects in the random group and 22.8% in the help seeking group fulfilling the criteria (see Table 1).

**Table 1:** Frequencies of diagnose-clusters, including most frequent specific diagnoses, in both subsamples at t1

ICD 10 chapter	help seekers	random
diagnosis	124 (78.5%)	56 (44.8%)
F0	1 (0.6%)	0 (0.0%)
F1	1 (0.6%)	1 (0.8%)
F2	2 (1.3%)	1 (0.8%)
F3	46 (29.1%)	16 (12.8%)
F32.1	14 (8.9%)	5 (4.0%)
F33.1	9 (5.7%)	4 (3.2%)
F33.2	12 (7.6%)	2 (1.6%)
F4	65 (41.1%)	31 (24.8%)
F43.1	36 (22.8%)	22 (17.6%)
F43.2	14 (8.9%)	7 (5.6%)
F5	8 (5.1%)	5 (4.0%)
F6	1 (0.6%)	2 (1.6%)
no diagnosis	34 (21.5%)	69 (55.2%)
total	158 (100%)	125 (100%)

Note. F0 = Organic, including symptomatic, mental disorders. F1 = Mental and behavioural disorders due to psychoactive substance use. F2 = Schizophrenia, schizotypal and delusional disorders. F3 = Mood (affective) disorders. F32.1 = Moderate depressive episode. F33.1 = Recurrent depressive disorder, current episode moderate. F33.2 = Recurrent depressive disorder, current episode severe without psychotic symptoms. F4 = Neurotic, stress-related and somatoform disorders. F43.1 = Posttraumatic stress disorder. F43.2 = Adjustment disorders. F5 = Behavioural syndromes associated with physiological disturbances and physical factors. F6 = Disorders of adult personality and behavior

**Table 2:** Relative frequencies of F3 and F4 diagnoses in both subsamples at t1

psychiatric disorder	help-seekers *n* = 124	random *n* = 56
F3	45 (36.3%)	16 (28.6%)
F31.0	1 (0.8%)	0 (0.0%)
F32.0	1 (0.8%)	3 (5.4%)
F32.1	14 (11.3%)	5 (8.9%)
F32.2	6 (4.8%)	1 (1.8%)
F32.3	1 (0.8%)	0 (0.0%)
F33.1	9 (7.3%)	4 (7.1%)
F33.2	12 (9.7%)	2 (3.6%)
F33.3	0 (0%)	1 (1.8%)
F34.1	2 (1.6%)	0 (0%)
F4	65 (52.4%)	31 (55.4%)
F41.0	8 (6.5%)	1 (1.8%)
F41.1	3 (2.4%)	0 (0%)
F41.2	1 (0.8%)	1 (1.8%)
F43.0	1 (0.8%)	0 (0%)
F43.1	36 (39.3%)	22 (29%)
F43.2	14 (11.3%)	7 (12.5%)
F44.0	1 (0.8%)	0 (0%)
F45.8	1 (0.8%)	0 (0%)

Note. F31.0 = Bipolar affective disorder, current episode hypomanic. F32.0 = Mild depressive episode. F32.1 = Moderate depressive episode. F32.2 = Severe depressive episode without psychotic symptoms. F32.3 = Severe depressive episode with psychotic symptoms. F33.1 = Recurrent depressive disorder, current episode moderate. F33.2 = Recurrent depressive disorder, current episode severe without psychotic symptoms. F33.3 = Recurrent depressive disorder, current episode severe with psychotic symptoms. F34.1 = Dysthymia. F41.0 = Panic disorder (episodic paroxysmal anxiety). F41.1 = Generalized anxiety disorder. F41.2 = Mixed anxiety and depressive disorder. F43.0 = Acute stress reaction. F43.1 = Posttraumatic stress disorder. F43.2 = Adjustment disorders. F44.0 = Dissociative amnesia. F45.8 = Other somatoform disorders. F51.0 = Nonorganic insomnia

Help-seekers also showed significantly more psychiatric symptoms compared to the random group across all scales of the BSI (see fig. 2), as well as the MADRS, and the PSQI (ANOVA all *p* < .000). These differences persisted after excluding all individuals without a psychiatric diagnosis from the analysis. In external assessment, at least temporary thoughts of suicide were documented in 26% of the help-seeking group and in 6% of the random group (*p* < .000). Likewise, the number of potentially traumatizing events in an individual’s previous history reported in ETI was 1.1 in the random sample and almost double this number in the help-seeking group (1.9, *p* < .000).

In the help-seeking group, 50.6% received a drug prescription from the psychiatrist as compared to 25.6% in the random group. Most of these prescriptions were for mild sedatives and antidepressants. 18 individuals were referred to psychiatric inpatient treatment, 13 to psychiatric outpatient, and 18 to non-psychiatric outpatient treatment.

### Follow-up (t2)

103 individuals were invited for a follow-up examination. For practical reasons, those that had moved away over 100 km from the study center in Nuremberg, Germany, were excluded from the follow-up. By the end of the study, 34 subjects (11 from the help-seeking group, 23 from the random group, age 32.50 ± 11.14 years) could be identified and examined. When controlling for group and gender, the follow-up sample did not differ significantly from the rest of the initial sample regarding symptoms on the BSI, MADRS or PSQI.

The average time lag between t1 and t2 was 214.6 ± 86.4 days. There were no significant differences between former help seekers and members of the random group regarding sociodemographic characteristics.

The total amount of psychiatric diagnoses decreased from 25 at t1 to 13 at t2. At t1, 41.2% had been diagnosed with an affective disorder from the F3 chapter of the ICD 10. At t2, 8.8% fit the criteria of such a disorder. All 7 cases of severe depressive episodes (F32.2, F33.2) had receded. Diagnoses of trauma and stress-related disorders stayed at 20.6% at both points in time. However, there was a shift, and arguably an increase, in severity: while there were four cases of adjustment disorder less, four new cases of PTSD were diagnosed (see Table 2). All three initial cases of non-organic insomnia either persisted or shifted to a more severe diagnose at follow-up.

More than half of the sample (*n* = 19) reported having been in contact with the public health system since t1, either receiving medical treatment (12), prescription drugs (2), psychotherapy (2), or other forms of therapy and counseling (3) (Table 3).

**Table 3:** Overview of psychiatric diagnoses at t1 and t2 in the follow-up sample. *N* = 34

diagnosis	t1	t2
F3	14	3
F32.1	4	1
F32.2	3	0
F33.1	3	1
F33.2	4	0
F34.1	0	1
F4	7	7
F43.1	3	7
F43.2	4	0
F51.0	3	3
F60.8	1	0
none	9	21

Note. F32.1 = Moderate depressive episode. F32.2 = Severe depressive episode without psychotic symptoms. F33.1 = Recurrent depressive disorder, current episode moderate. F33.2 = Recurrent depressive disorder, current episode severe without psychotic symptoms. F34.1 = Dysthymia. F43.1 = Posttraumatic stress disorder. F43.2 = Adjustment disorders. F51.0 = Nonorganic insomnia. F60.8 other specific personality disorder

## Discussion

Our results show a high prevalence of psychiatric disorders in asylum seekers with a significantly higher prevalence in the help-seeking group of patients as opposed to the random group. The most frequent primary diagnosis in both groups was PTSD (F43.1). Compared to the research provided by Wittchen and colleagues [30], the prevalence is clearly higher in our random sample than in the European population (17.6% vs. 2.9%). This might be explained by the potentially traumatic circumstances that may have led this population to seek asylum in the first place, in combination with post migration stress [31–33]. The second most frequent diagnosis found was a depressive episode (light to severe) followed by insomnia. When we combine the prevalence of stress-related and affective disorders, the results are similar to those shown in the research on refugees by Lindert and colleagues [5] (37.6% vs. 40%). In total, the prevalence rates in our random sample falls into the mid-range of values reported in literature on this topic [4, 12, 14–16].

From the collected data we conclude that asylum seekers tend to be more vulnerable to psychiatric illness. This reflects the assumption by Lindert and colleagues [5] that within the heterogeneous group of migrants there is a higher prevalence of depression and anxiety in asylum seekers than in other groups, such as labor migrants.

From t1 to t2, the prevalence of PTSD increased without evident additional traumatic events. According to the diagnostic criteria for PTSD in ICD 10 [24], there is a time latency of several weeks or months for the manifestation of the main symptoms. These results suggest that asylum seekers’ psychiatric diagnoses soon after arrival should be recorded carefully and that examination should be repeated after six months. Otherwise, PTSD symptoms might remain undiscovered and grow even more severe. Considering the diagnoses of depression, the disease receded in 11 of 13 cases. We assume that the improvement of social circumstances and living conditions after leaving the crowded admission center might be contributing to this, as only five subjects in the follow-up sample had received some sort of psychotherapy or counselling. The authors conclude that the psychological and psychiatric treatment of mentally ill asylum seekers after discharge from the admission center is insufficient.

The typical subjects in the help-seeking group were Russian women of Christian denomination with previous migration experience. In the random group, there was a higher proportion of male asylum seekers of Muslim denomination coming mainly from Iraq and Iran, of which 45% fulfilled the criteria for a psychiatric diagnosis. They, however, were not seeking help. The cause for this appears to be due to gender as well as cultural, ethnic and religious differences in how psychiatric symptoms are understood and ultimately expressed. Moreover, they could be a starting point for the targeted treatment of risk groups. In order for public health systems to cope with this problem, the authors suggest psychoeducation and information for asylum seekers with the aim to relieve stigmatization from mental illness.

### Implications

From our perspective, a multi-professional and multilingual team with a background in cultural psychiatry is necessary to optimally diagnose and treat a population as heterogenous and burdened as asylum seekers. In the help-seeking group, 21.5% did not fulfill the criteria for a psychiatric illness. We assume that they may have suffered from psychological problems such as anxiety or sleep problems, but their symptoms were not distinct enough to fulfil the criteria for a psychiatric diagnosis. The second reason could be simulation of symptoms wishing to be granted asylum, which we can only assume. We believe, however, that by having two examiners (a psychiatrist and a psychologist) we were able to minimize false positives.

The diagnosis of recurrent depression (F33) was at 9.7% of the random and 17% of the help-seeking group. This means that a high number of asylum seekers are likely to have suffered from depression in the past, which could impact on individual help-seeking behavior.

The fourth most frequent psychiatric diagnosis in our study was insomnia. In order to be able to make a difference between insomnia and insomnia symptoms, we asked for the severity of sleep difficulties. Usually, the subject reported that their insomnia bothered them much more than the depressive or anxious mood and that better sleep would also improve their mood [34]. Our experience was that for some asylum seekers words such as *psychiatry*, *psychology*, or *stress* were not familiar. However, asking how they slept opened the door for better understanding of their psychological status. For this reason, sleep could provide an effective avenue for treatment [35].

We did not diagnose an ‘Enduring personality change after catastrophic experience’ in any of the participants – one explanation for this fact was that not enough time had passed from the time of trauma.

Our final recommendation for further research on the evaluation of mental disorders in the population of asylum seekers is to always include a randomized control group – this is especially important to help gain representative data for this very diverse population.

### Limitations and weaknesses

The random sample could have been structured differently so as to mirror the sociodemographic make-up of the residents at the ZAE Zirndorf more closely and to be more representative of this unit. At the same time, however, the high fluctuation of residents might have counteracted this approach. This means that the random sample only represents a snapshot of the situation at a given moment in time. A more long-term comparison including the statistics for the years 2011 and 2012 might provide further insights in this respect. What remains unclear is whether the site of interview had an impact on help seeking behavior because all of our interviews occurred onsite at the ZAE. Future studies should investigate help seeking behavior in other locations, such as hospitals. Finally, due to administrative and economic reasons, the follow-up group was a lot smaller than intended and may not have produced entirely representative results.

## Conclusions

On the basis of current research, we recommend the establishment of outpatient psychiatric-psychological services for those who need treatment as well as short psychiatric screenings for all asylum seekers immediately upon their arrival in the host country. Screening and supportive consultation should be available as part of the first wave of medical screening/treatment with an option for pharmacotherapy if appropriate and necessary. Psychotherapeutic treatments could be offered as a second step in a hierarchy of treatment model. With regard to post migration stress, a helpful step would be to reduce the temporal duration of the application procedure for asylum status - this should also include limiting the time spent in an admission center.

## Abbreviations

BSI: Brief Symptom Inventory
ETI: Essen Trauma Inventory
ICD 10: International Classification of Diseases, 10th revision
MADRS: Montgomery Asberg Depression Rating Scale
MINI: Mini-International Neuropsychiatric Interview
PSQI: Pittsburg Sleep Quality Index
PTSD: Posttraumatic Stress Disorder
ZAE: Admission Center for Asylum Seekers (“*Zentrale Aufnahmeeinrichtung*”)
